# Mentalizing Across the Psychosis Continuum in Adolescence and Young Adulthood: A Systematic Review and Narrative Synthesis

**DOI:** 10.1093/schbul/sbaf095

**Published:** 2025-07-10

**Authors:** George Salaminios, Astra Hazlitt, Peter Fonagy, Martin Debbané, Tobias Nolte

**Affiliations:** Research Department of Clinical, Educational and Health Psychology, University College London, London WC1E 6BT, United Kingdom; Research Department, British Association for Counselling and Psychotherapy, Lutterworth LE17 4HB, United Kingdom; Research Department of Clinical, Educational and Health Psychology, University College London, London WC1E 6BT, United Kingdom; Southwark Child and Adolescent Carelink Sevice, South London and Maudsley NHS Foundation Trust, London SE15 5LJ, United Kingdom; Research Department of Clinical, Educational and Health Psychology, University College London, London WC1E 6BT, United Kingdom; Anna Freud, London N1 9JH, United Kingdom; Research Department of Clinical, Educational and Health Psychology, University College London, London WC1E 6BT, United Kingdom; Developmental Clinical Psychology Research Unit, Faculty of Psychology and Educational Sciences, University of Geneva, Geneva 1211, Switzerland; Research Department of Clinical, Educational and Health Psychology, University College London, London WC1E 6BT, United Kingdom; Anna Freud, London N1 9JH, United Kingdom

**Keywords:** mentalization, schizotypy, first episode psychosis, CHR, reflective functioning, ToM

## Abstract

**Background and Hypothesis:**

Mentalizing difficulties have consistently been identified in adult samples across the psychosis continuum. However, the links between mentalizing and psychosis expression during the critical period spanning from adolescence to young adulthood, when the earliest signs of psychosis commonly emerge, remain less clear. The current review aims to synthesize and evaluate existing findings on the presence and role of mentalizing dysfunction at each stage of the psychosis continuum in adolescent and young adult samples.

**Study Design:**

Electronic databases were used to identify empirical articles examining the links between mentalizing and psychosis expression in community, clinical-high risk for psychosis (CHR-P), first episode psychosis (FEP), and clinical psychosis samples aged 10-25 years. A narrative synthesis approach was used to integrate and evaluate the study findings.

**Results:**

Twenty-six eligible studies were identified with a combined sample of 4391 individuals. The synthesis of findings indicates mixed evidence on the links between mentalizing and nonclinical psychotic manifestations in community samples. Most studies with CHR-P samples suggest that mentalizing difficulties are present during the psychosis prodrome and may contribute to its clinical progression. Studies among adolescents and young adults with FEP and those with a previous diagnosis of psychotic disorders indicate that mentalizing impairments are present at the point of conversion and early course of the illness.

**Conclusions:**

The findings of the review suggest that mentalizing difficulties can be observed across the psychosis continuum in adolescence and young adulthood, particularly during the prodromal and clinical stages and may constitute valid early intervention targets.

## Introduction

Contemporary research suggests that clinical psychosis is a neurodevelopmental disorder that commonly emerges during late adolescence/young adulthood and is preceded by premorbid and prodromal signs manifesting primarily within the domains of perception, cognition, and interpersonal functioning.^1^ On this basis, most current clinical conceptualizations view psychosis as expressed along a continuum, ranging from nonclinical and relatively stable trait abnormalities (ie, schizotypal personality traits, psychotic-like experiences), to prodromal clinical high-risk manifestations (CHR-P, ie, brief and attenuated psychotic symptoms), and finally to the pervasive reality distortions that mark the transition to the first episode of psychosis (FEP).^2,3^

Importantly, the progression from premorbid and CHR-P manifestations to FEP, as well as the duration of untreated psychosis, have been linked to adverse outcomes in the domains of social, interpersonal, and occupational functioning that often persist despite symptomatic improvement following psychological or pharmacological interventions.^4^ For this reason, the focus of treatment efforts has been shifting toward an early intervention approach, seeking to identify and treat emerging psychosis during its earliest stages of expression in adolescence and young adulthood.^5^ However, the psychological factors that contribute to, or mitigate psychosis development during the critical period spanning from adolescence to young adulthood and may influence the transition from premorbid or prodromal manifestations to clinically diagnosable presentations remain incompletely understood. Several studies suggest that early intervention might improve clinical and functional outcomes,^6,7^ thus further underscoring the need to elucidate the psychological functions that should be targeted early to attenuate the progression of emerging psychosis.^8^

An important psychological factor modulating the expression of psychosis across its developmental continuum may be mentalizing—the capacity to perceive or interpret one’s own and others’ behaviors as being driven by intentional mental states, such as thoughts and feelings.^9–11^ Mentalizing constitutes a multifaceted construct that encompasses a range of higher order cognitive processes involved in mental state understanding and affects regulation.^12^ These enable individuals to construe representational models of human behavior and inner experience in order to navigate the complexity of social interactions, as well as reflect on and regulate their own thinking and feeling states.^9^ Given the role of mentalizing in affect regulation^13^ and its contribution to supporting adaptive functioning within interpersonal contexts, it has been hypothesized that sustaining mentalizing abilities may promote recovery in people suffering with psychotic disorders, as well as confer resilience against transition to clinical illness among those who are at increased risk.^10^

Mentalizing impairments have consistently been associated with symptomatic and functional outcomes in people diagnosed with psychotic disorders.^14^ Furthermore, previous studies suggest that first-episode sufferers and people that meet CHR-P criteria perform poorly in multiple domains of mentalizing, such theory of mind (ToM) and emotion recognition.^15^ However, these often involve adult samples that are beyond the critical period during which the first non-clinical and clinical signs of psychosis commonly emerge. Research focusing on adolescent and young adult samples can further elucidate our understanding regarding the role of mentalizing in the early course of psychosis expression, with important implications for early intervention treatment.

A number of studies have assessed the links between mentalizing and psychosis expression among adolescents and young adults exhibiting premorbid, prodromal, or clinical manifestations. These have reported inconsistent findings, with wide variations in methodological designs, including data analytic methods and measures used to assess mentalizing abilities and psychosis expression. To date, no attempts have been made to systematically review, synthesize, and evaluate existing research examining the presence and role of mentalizing dysfunction across the psychosis continuum, specifically during the critical developmental period spanning from adolescence to young adulthood. Therefore, the overarching aim of the current systematic review is to assess whether mentalizing difficulties are observable at each stage of the psychosis continuum during adolescence and young adulthood. More specifically, the current systematic review will use a narrative synthesis approach to integrate and evaluate findings from studies that have examined mentalizing abilities and psychosis expression among adolescents and young adults, including community, CHR-P, FEP, and clinical psychosis samples.

## Methods

This systematic review follows the Preferred Reporting Items for Systematic reviews and Meta-Analyses (PRISMA) recommendations.^16^ In line with PRISMA guidelines, the following sections describe the methods used to search, appraise, and synthesize the studies included in the review. The systematic review protocol has been registered in PROSPERO (CRD42024572811).

### Search Strategy

Because mentalizing is a multifaceted construct that encompasses a wide range of related concepts (eg, “empathy,” “metacognition,” “affect consciousness”),^12^ we chose to narrow the search terms on the concepts of “mentalization,” “theory of mind” (ToM), and “reflective functioning.” Theory of mind was included in the review as a search term because, to date, mentalizing abilities in psychosis have primarily been assessed through the use of ToM tasks.^10^ In addition, reflective functioning was included as it has been cited as the operationalization, for assessment and measurement purposes, of the psychological processes underpinning mentalizing.^17,18^

We aimed to identify studies examining psychosis at each stage of its developmental continuum during adolescence and young adulthood. We therefore searched for studies exploring mentalizing abilities in the following populations: (1) community adolescents/young adults assessed for schizotypal traits and/or psychotic experiences; (2) adolescents/young adults who meet CHR-P criteria, (3) adolescents/young adults presenting with FEP; and (4) adolescents/young adults with a previous diagnosis of a psychotic disorder.

Studies were identified from four electronic databases: PsycINFO, Embase, MEDLINE, and Web of Science. Each database was searched from inception until the 31st of July 2024. To support a thorough search, database subject heading searches were included for “mentalization,” “theory of mind,” “reflective functioning,” and “psychosis” on PsycINFO, Embase, and MEDLINE (Web of Science does not use subject headings).

The search terms, in addition to subject headings, were: (mentaliz* OR mentalis* OR (“theory of mind”) OR (“reflective function*”)) AND (psychosis OR psychotic OR schizo* OR hallucin* OR delusion* OR (“clinical high-risk”) OR (“ultra-high risk”) OR (“at-risk mental state”)) AND (adolescen* OR youth* OR (“young adult*”) OR (“emerging adult*”) OR (“young people”) OR student). The specific search queries used for each database are included in Supplementary Material.

### Screening and Selection Criteria

We aimed to identify studies investigating the links between mentalizing and psychosis expression among adolescent and young adult samples. Specific inclusion criteria were: (1) study population aged between 10 and 25 years; (2) study included a measure related to mentalizing (eg, reflective functioning, ToM); (3) study included a measure or group related to psychosis expression (eg, community sample assessed for psychotic-like experiences/schizotypal traits, CHR-P sample, FEP sample, clinical psychosis sample); (4) study analyzed mentalizing as related to psychosis expression (ie, group comparisons on measures of mentalizing, or the outcome of any associations tested between continuous measures of mentalizing and continuous measures of psychosis expression, or both).

Articles were excluded if they were: (1) non-empirical (eg, reviews, commentaries, theoretical papers, editorials, books/book chapters); (2) conference abstracts; (3) qualitative studies; and (4) case study or case series.

The search and selection processes were completed independently by two reviewers (A.H. and G.S.). First, the results retrieved from the search of the four databases were combined and duplicate studies were removed. Next, titles and abstracts of all studies were screened against the inclusion and exclusion criteria to identify potentially eligible studies. As a final step, the full texts of shortlisted studies were screened according to the same criteria.

### Data Extraction

Once all eligible studies were identified, two reviewers (A.H. and G.S.) independently assessed the full text of each research report. To compare study characteristics, information about sample size, study design, mean sample age/age range, gender distribution, study population (community, CHR-P, FEP, psychotic disorder), measures used to assess mentalizing and psychosis expression, as well as type of statistical analyses used to test the relation between mentalizing and psychotic features was extracted and integrated using a narrative approach.

In terms of findings, for each study we sought the outcome of any group comparisons made (eg, CHR-P group vs. nonclinical controls) on measures of mentalizing, and/or the outcome of any associations tested between continuous measures of mentalizing and continuous measures of psychotic expression (eg, schizotypal traits, psychosis symptoms), or both.

### Data Synthesis and Analysis

Due to the heterogeneity of studies and different methods used to assess psychosis expression and mentalizing, a narrative synthesis was chosen as the most suitable approach to integrate the study findings.^19^ For the purposes of the narrative synthesis, study findings were grouped according to study population, specifically as it pertains to their stage of psychosis expression (ie, community adolescent sample, clinical high risk for psychosis sample, first episode of psychosis sample, clinical psychosis sample). Grouping studies according to study population enabled us to explore whether mentalizing dysfunction is present across each different stage of psychosis expression during adolescence and young adulthood.

Two reviewers (A.H. and G.S.) independently conducted the quality assessment after reviewing the full text of each study. The quality of the evidence was assessed using the QualSyst tool for systematic reviews,^20^ a standardized tool that sets out criteria for evaluating primary research papers. The quantitative scoring tool used in this review consists of fourteen items (see supplementary material). Items 5, 6, and 7 were excluded as they pertain to interventional research, thus not applicable to the current review. The QualSyst manual describes how to assess/score each individual item and calculate the total score from the items.^20^ Each item is scored based on the degree to which the criterion is met (Yes = 2; Partially met = 1; No = 0). The total score for each paper is then calculated by summing the score of individual items and dividing it by the maximum possible sum score for all individual items. The maximum possible total score for each paper is 1, with higher scores indicating higher study quality. Overall, our quality assessment considered the following methodological domains: research questions and objectives, study design, selection bias, definitions and robustness of outcomes, sample size, data analytic methods, confounders, and reporting of results and conclusions.

## Results

### Study Selection

**Figure 1.**
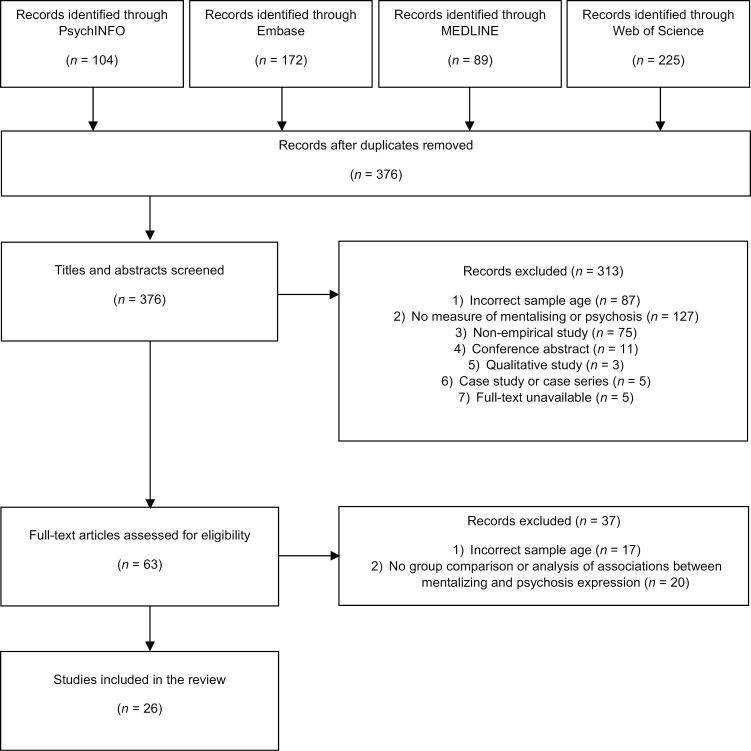
PRISMA Flow Diagram of Study Selection

As shown in Figure 1, the initial search identified 376 records after duplicates were removed, and 313 records were excluded from the review after screening titles and abstracts against the inclusion/exclusion criteria. From the 63 remaining studies that were shortlisted for full-text screening, 26 were included in the review after meeting all necessary inclusion criteria.

### Study Characteristics

The characteristics of the twenty-six studies included in the review are displayed in Table 1. Most studies used a cross-sectional design (*n* = 21, 81%),^21–29,31–33,35,37,40–46^ with the remaining ones employing longitudinal designs (*n* = 5, 19%).^30,34,36,38,39^ Three studies with a longitudinal design assessed the predictive value of mentalizing abilities in distinguishing between CHR-P participants who transitioned to FEP from those that did not.^30,36,39^

**Table 1.** Study Characteristics, *N* = 26 (Listed According to Publication Year).

Authors, Year	Design	*n*	Age (mean/range)	Male/Female (%)	Study Population	Mentalizing Measures	Mentalizing Dimensions Assessed	Psychosis Measure	Type of Statistical Analysis
Jahshan and Sergi (2007)^21^	Cross-sectional	92	20.1 (18-25)	41/59	Community	TASIT	Other-oriented Cognitive/affective	SPQ-Brief	Group differences
Fernyhough et al. (2008)^22^	Cross-sectional	828	20.3 (18-25)	39/61	Community	The Hinting Task; Cartoon Jokes Task	Other-oriented Cognitive	O-LIFE-Brief; PIQ	Group differences; regression
Barragan et al. (2011)^23^	Cross-sectional	72	14.5 (13-16)	54/46	Community	Strange Stories Task	Other-oriented Cognitive/affective	CAPE; O-LIFE	Regression
Thompson et al. (2012)^24^	Cross-sectional	100	19.6 (15-25)	49/51	CHR (*n* = 30) FEP (*n* = 40) HC (*n *= 30)	The Hinting Task; Cartoon Jokes Task	Other-oriented Cognitive	CAARMS; SANS	Group differences
Korver-Nieberg et al. (2013)^25^	Cross-sectional	110	16.7 (13-18)	64/36	FEP (*n *= 32) HC (*n* = 78)	Perspective Taking Task	Other-oriented Cognitive	PANSS; CAPE; GPTS	Group differences; regression
Langdon et al. (2014)^26^	Cross-sectional	42	20.8 (17-25)	93/7	FEP (*n* = 23) HC (*n *= 19)	Cartoon Jokes Task; Strange Stories Task; Story Sequencing Task	Other-oriented Cognitive/affective	DIP; SANS	Group differences; correlation
Canli et al. (2015)^27^	Cross-sectional	44	16.5 (15-17)	59/41	Community	EQ; Faux Pas Task; The Hinting Task	Other-oriented Cognitive/affective	MIS	Group differences
Clemmensen et al. (2015)^28^	Cross-sectional	1630	11.6 (11-12)	781/849	Community	ToM Storybook Frederik	Other-oriented Cognitive/affective	K-SADS	Regression
Bourgou et al. (2016)^29^	Cross-sectional	24	14.7 (13-17)	54/46	Psychotic disorder (*n = 12)* HC (*n *= 12)	Moving Shapes Paradigm	Other-oriented Cognitive	SCID-5; PANSS	Group differences; correlation
Zhang et al. (2016)^30^	Longitudinal	82	17.9 (15-20)	49/51	CHR (*n *= 40) HC (*n* = 42)	RMET	Other-oriented Affective	SIPS	Group differences
Li et al. (2017)^31^	Cross-sectional	70	16.4 (13-18)	57/43	Psychotic disorder (*n* = 35) HC (*n* = 35)	Faux Pas Task; Yoni Task	Other-oriented Cognitive/affective	PANSS	Group differences; correlation
Bartholomeusz et al. (2018)^32^	Cross-sectional	36	20.4 (15-25)	47/53	FEP (*n* = 14) HC (*n* = 22)	The Hinting Task; Story Sequencing Task	Other-oriented Cognitive	BPRS; SANS	Group differences
Ilzarbe et al. (2019)^33^	Cross-sectional	68	18.0 (15-20)	43/57	Psychotic disorder (*n* = 27) HC (*n* = 41)	RMET	Other-oriented Affective	K-SADS; PANSS	Group differences
Steenhuis et al. (2019)^34^	Longitudinal	157	18.9 (18-19)	43/57	Community	Theory of Mind Storybook Frank	Other-oriented Cognitive/affective	CAPE	Group differences; regression
Vargas et al. (2019)^35^	Cross-sectional	50	19.8 (13-24)	54/46	CHR (*n* = 24) HC (*n* = 26)	Short Story Task	Other-oriented Cognitive/affective	SCID-5; SIPS	Group differences; correlation
Boldrini et al. (2020)^36^	Longitudinal	110	16.9 (12-25)	40/60	CHR (*n* = 57) Clinical controls (*n* = 53)	RFS of AAI	Self/other Cognitive/affective	SIPS	Group differences; correlation; regression
Shi et al. (2020)^37^	Cross-sectional	46	13.4 (10-16)	58/42	Psychotic disorder (*n* = 20) HC (*n* = 26)	GEM	Other-oriented Affective	SCID-5; PANSS	Group differences; correlation
Carey et al. (2021)^38^	Longitudinal	103	20.9 (19-25)	49/51	Community	RMET	Other-oriented Affective	K-SADS; SCID-5	Group differences
Ilzarbe et al. (2021)^39^	Longitudinal	86	15.5 (12-18)	33/67	CHR (*n* = 50) HC (*n* = 86)	RMET	Other-oriented Affective	SIPS	Group differences
Salaminios et al. (2021)^40^	Cross-sectional	105	15.7 (12-19)	51/49	Community	RFQ	Self/other Cognitive/affective	SPQ; YSR	Correlation; regression
Kong et al. (2021)^41^	Cross-sectional	56	20.4 (16-23)	68/32	CHR (*n* = 28) HC (*n* = 28)	ToM-PST IRI	Other-oriented Cognitive/affective	SCID-5	Group differences
El Ray et al. (2022)^42^	Cross-sectional	90	22.7 (15-25)	61/39	Psychotic disorder (*n* = 30) HC (*n* = 30)	RMET; Ekman 60-Faces Test	Other-oriented Affective	SCID-5; PANSS	Group differences; correlation
Saglam et al. (2022)^43^	Cross-sectional	156	17.3 (13-22)	63/37	Psychotic disorder (*n* = 106) HC (*n* = 50)	RMET; Faux Pas Task	Other-oriented Cognitive/affective	K-SADS; PANSS	Group differences
Shen et al. (2022)^44^	Cross-sectional	45	13.3 (10-15)	47/53	Psychotic disorder (*n* = 20) HC (*n* = 25)	RMET	Other-oriented Affective	SCID-5	Group differences
Salaminios et al (2023)^45^	Cross-sectional	87	19.3 (14-23)	46/54	Community	RFQ	Self/other Cognitive/affective	SPI-A/SPI-CY	Regression
Sardella et al (2023)^46^	Cross-sectional	102	18.1 (16-19)	51/49	CHR (*n* = 51) HC (*n* = 51)	RFQ-B	Self/other Cognitive/affective	SCID-5	Group differences

HC = Healthy Control, FEP = First Episode Psychosis, CHR = Clinical High Risk, TASIT = The Awareness of Social Inference Test, EQ = Empathy Quotient, RMET = Reading the Mind in the Eyes Test, RFS = Reflective Functioning Scale, AAI = Adult Attachment Interview, GEM = Griffith Empathy Measure, RFQ = Reflective Functioning Questionnaire, ToM-PST = Theory of Mind Picture Stories Task, SPQ = Schizotypal Personality Questionnaire, O-LIFE = Oxford-Liverpool Inventory of Feelings and Experiences, PIQ = Persecutory Ideation Questionnaire, CAPE = Community Assessment of Psychic Experiences, SIPS = Structured Interview for Prodromal Symptoms, CAARMS = Comprehensive Assessment of At Risk Mental States, SANS = Scale for the Assessment of Negative Symptoms, PANSS = Positive and Negative Syndrome Scale, GPTS = Green Paranoid Thought Scale, DIP = Diagnostic Interview for Psychoses, MIS = Magical Ideation Scale, SCID-5 = Structured Clinical Interview for the Diagnostic and Statistical Manual of Mental Disorders, Fifth Edition, YSR = Youth Self-Report, BPRS = Brief Psychiatric Rating Scale, K-SADS = Kiddie Schedule for Affective Disorders and Schizophrenia.

Sample sizes varied across the studies, ranging from *n* = 24 in a study comparing adolescents with early-onset schizophrenia with nonclinical controls,^29^ to *n* = 1630 in a study that included a large community sample of young people.^28^ The mean age of the reported samples ranged from 11.6 to 22.7 years. The mean age across all studies was 17.7. There was wide variability in the age ranges between studies, with the combined age range covering 10-25 years. All studies used a mixed gender sample.

Study populations provided a range of psychotic features across the continuum. Nine studies assessed psychosis expression in community adolescents and young adults. Seven studies tested mentalizing abilities in adolescents and young adults meeting criteria for CHR-P. Four studies included samples of adolescents and young adults presenting with FEP and six studies assessed adolescents and young adults with a previous diagnosis of a psychotic disorder.

The type of statistical analyses used to test the role of mentalizing in psychosis expression varied between studies. Twelve studies split their sample into subgroups based on the severity of psychosis expression and analyzed group differences on mentalizing abilities (eg, FEP compared with nonclinical controls, or high schizotypy group compared with low schizotypy group). Three studies used regression or correlation analyses to test associations between continuous measures of mentalizing and psychosis expression. The remaining eleven studies utilized both methods, testing both group differences in mentalizing abilities, as well as associations between continuous measures of mentalizing and psychosis expression.

### Study Measures

Across the twenty-six studies included in this review, twenty-one different measures were used to operationalize mentalizing and seventeen different measures were used to assess psychosis expression. Tables 2 and 3 provide details on the measures used to assess mentalizing and psychosis expression, respectively.

**Table 2.** Measures of Mentalizing Used by Included Studies (Listed Alphabetically).

Measure	Type of measure	Brief description of measure	Used by
Cartoon Jokes Task^47^	Experimental task (picture-based)	A series of visual cartoons are shown, in which understanding the joke depends on being able to infer a character’s mental state. Participants are scored on ability to explain the joke with direct reference to the character’s mental state.	Fernyhough et al. (2008) Thompson et al. (2012) Langdon et al. (2014)
Empathy Quotient (EQ)^48^	Self-report questionnaire	60-item questionnaire measuring empathy. It assesses ability to identify the intentions of another person, predict another person’s behavior, and experience an emotion triggered by another person.	Canli et al. (2015)
Ekman 60-Faces Test^49^	Experimental task (picture-based)	The faces of 10 different characters are shown, with each displaying six basic emotions (happiness, sadness, disgust, fear, surprise, and anger). Participants are scored on ability to correctly recognize the emotion.	El Ray et al. (2022)
Faux Pas Test^50^	Experimental task (verbal)	Participants listen to a series of social stories in which one of the characters makes a “faux pas.” They are scored on ability to detect and explain what should not have been said and why, referencing the characters’ mental states.	Canli et al. (2015) Li et al. (2017) Saglam et al. (2022)
Griffith Empathy Measure (GEM)^51^	Parent-report questionnaire	A questionnaire in which parents respond on a nine-point Likert scale to 18 items assessing their children’s level of cognitive and affective empathy, such as “My child rarely understands why other people cry.”	Shi et al. (2020)
Interpersonal Impulsivity Index (IRI)^52^	Self-report Questionnaire	28-item questionnaire measuring empathy. It is comprised of four subscales with 7 items each: empathic concern, perspective taking, personal distress, fantasy	Kong et al. (2021)
Moving Shapes Paradigm^53^	Experimental task (visual)	12 animations of moving shapes are shown, reflecting three types of movement: random, goal-directed, and mentalizing interactions. Participants are scored on their ability to describe the actions, intentions, and implied mental states of the shapes.	Bourgou et al. (2016)
Perspective Taking Task^h54^	Experimental task (visual)	Computerized task in which a “director” instructs participants to move objects on a set of shelves. Some objects are invisible to the director. Participants are scored on ability to consider the director’s ignorance to certain objects when making movements.	Korver-Nieberg et al. (2013)
Reading the Mind in the Eyes Test (RMET)^55^	Experimental task (picture-based)	Images of various people’s eye regions depicting different expressions are shown. Participants are scored on their choice from four options of which word best describes the mental state reflected in the eyes.	Zhang et al. (2016) Ilzarbe et al. (2019) Carey et al. (2021) Ilzarbe et al. (2021) El Ray et al. (2022) Saglam et al. (2022) Shen et al. (2022)
Reflective Functioning Questionnaire (RFQ)^18^	Self-report questionnaire	54-item self-report questionnaire measuring mentalizing by evaluating the respondent’s certainty (RFQc scale) and uncertainty (RFQu scale) about the mental states of themselves and others.	Salaminios et al. (2021) Salaminios et al. (2023)
Reflective Functioning Questionnaire—Brief (RFQ-B)^56^	Self-report questionnaire	Shortened 8-item version of RFQ, comprised of the same two scales measures respondents’ certainty and uncertainty about the mental states of themselves and others	Sardella et al. (2023)
Reflective Functioning Scale (RFS) of the Adult Attachment Interview (AAI)^57^	Interview coding scale	Responses from the Adult Attachment Interview are coded to assess the participants ability to understand potential mental states underlying their own and others’ attachment-related behavior.	Boldrini et al. (2020)
Short Story Task^58^	Experimental task (verbal)	After reading a short story about a romantic couple, participants are scored on 14 questions assessing their explicit and spontaneous mental state inferences of the characters, as well as on their comprehension of story facts.	Vargas et al. (2019)
Story Sequencing Task^m59^	Experimental task (picture-based)	Participants are shown four picture cards of a social interaction in an incorrect order and are scored on ability to rearrange them correctly to depict a logical sequence of events, drawing on information about the inferred mental states of the characters.	Langdon et al. (2014) Bartholomeusz et al. (2018)
Strange Stories Task^60^	Experimental task (verbal)	Participants read a set of short stories about different characters. After each story they are scored on their responses to questions requiring an inference about the character’s thoughts, feelings, and intentions.	Barragan et al. (2011) Langdon et al. (2014)
The Awareness of Social Inference Test (TASIT)^61^	Experimental task (video-based)	After viewing separate videotapes involving actors, participants are scored on their selection of emotions expressed by actors, and on their response to Yes/No questions as to mental states of the actors.	Jahshan and Sergi (2007)
The Hinting Task^62^	Experimental task (verbal)	Participants read short passages involving an interaction between two characters, at the end of which one character verbally drops a hint. They are then scored on ability to infer and explain the character’s real intentions behind the indirect speech.	Fernyhough et al. (2008) Thompson et al. (2012) Canli et al. (2015) Bartholomeusz et al. (2018)
Theory of Mind (ToM) Picture Stories Task (ToM-PST)^63^	Experimental task (picture-based)	Participants are presented with six cartoon picture sets, with four pictures each, and are first asked to arrange the cards into a logical sequence of events. In the second step, after cards are sequenced correctly, participants are asked to infer the cognitive and affective mental states of the characters.	Kong et al. (2021)
Theory of Mind (ToM) Storybook Frank^q64^	Experimental task (picture-based and verbal)	A storybook with 20 pictures is read aloud. Participants are scored on their responses to questions about their comprehension of the situation and their understanding of the characters’ mental states.	Steenhuis et al. (2019)
ToM Storybook Frederik (ToM-F)^65^	Experimental task (picture-based and verbal)	A storybook with 16 pictures is read aloud. Participants are scored on their responses to questions testing second-order false belief, white lie, irony and faux pas.	Clemmensen et al. (2015)
Yoni Task^66^	Experimental task (visual)	Computerized task in which participants are scored on ability infer which of four items a cartoon character “Yoni” is referring to in a verbal prompt, based on the available verbal, facial and eye gaze cues.	Li et al. (2017)

**Table 3.** Measures of Psychosis Expression Used in Included Studies (Listed Alphabetically).

Measure	Type of measure	Brief description of measure	Used by
Brief Psychiatric Rating Scale (BPRS)^67^	Clinician-rated scale	A questionnaire in which clinicians respond on a seven-point Likert scale to 18 items assessing a patient’s psychiatric symptoms including grandiosity, suspiciousness, hallucinations, and unusual thought content.	Bartholomeusz et al. (2018)
Comprehensive Assessment of At-Risk Mental States (CAARMS)^68^	Semi-structured interview	Semi-structured interview guide designed to be used by clinicians to assess the psychopathology indicative of someone developing a FEP and to identify young people who meet the criteria for being at CHR-P.	Thompson et al. (2012) Bartholomeusz et al. (2018)
Community Assessment of Psychic Experiences (CAPE)^69^	Self-report questionnaire	42-item self-report questionnaire developed to measure positive, negative, and depression symptom dimensions in the general population. Participants respond on a four-point Likert scale indicating frequency of symptoms	Barragan et al. (2011) Korver-Nieberg et al. (2013) Steenhuis et al. (2019)
Green Paranoid Thought Scale (GPTS)^70^	Self-report questionnaire	32-item self-report questionnaire measuring two dimensions of paranoid thinking: ideas of reference and ideas of persecution. Participants respond on a five-point Likert scale indicating frequency of symptoms.	Korver-Nieberg et al. (2013)
Kiddie Schedule for Affective Disorders and Schizophrenia (K-SADS)^71^	Semi-structured interview	Semi-structured interview designed to be used by clinicians to assess symptoms of affective and psychotic disorders in children and adolescents aged 6-18 years, according to the DSM-5 diagnostic criteria.	Ilzarbe et al. (2019) Carey et al. (2021) Saglam et al. (2022) Clemmensen et al. (2015)
Magical Ideation Scale (MIS)^72^	Self-report questionnaire	30-item self-report questionnaire designed to measure psychosis proneness by exploring beliefs related to magical influences, such as telepathy, astronomy, good luck charms and psychic energy. All items are rated on a True/False response format.	Canli et al. (2015)
Oxford-Liverpool Inventory of Feelings and Experiences (O-LIFE)^73^	Self-report questionnaire	104-item self-report questionnaire designed to measure schizotypy. It is comprised of four subscales: unusual experiences, introvertive anhedonia, cognitive disorganization, and impulsive non-conformity. Items are rated on a Yes/No response format.	Barragan et al. (2011)
Oxford-Liverpool Inventory of Feelings and Experiences Brief (O-LIFEB)^74^	Self-report questionnaire	Shortened 30-item version of the O-LIFE. It comprises of two subscales related to positive and negative schizotypy features: unusual experiences and introvertive anhedonia. Items are rated on a Yes/No response format.	Fernyhough et al. (2008)
Positive and Negative Syndrome Scale (PANSS)^75^	Semi-structured interview and clinician-rated scale	Clinicians respond on a seven-point Likert scale to 30 items assessing the severity of psychotic symptoms in patients with schizophrenia. It is comprised of three symptom subscales: positive, negative, and general psychopathology.	Korver-Nieberg et al. (2013) Bourgou et al. (2016) Li et al. (2017) Ilzarbe et al. (2019) Shi et al. (2020) El Ray et al. (2022) Saglam et al. (2022)
Persecutory Ideation Questionnaire (PIQ)^76^	Self-report questionnaire	10-item self-report questionnaire designed to measure persecutory delusion-like beliefs. Participants rate items on a five-point Likert scale indicating how true each statement is when applied to themselves.	Fernyhough et al. (2008)
Scale for the Assessment of Negative Symptoms (SANS)^77^	Clinician-rated scale	A questionnaire in which clinicians respond on a six-point Likert scale to 25 items measuring the severity of negative psychotic symptoms in patients with schizophrenia.	Thompson et al. (2012) Langdon et al. (2014) Bartholomeusz et al. (2018)
Structured Clinical Interview for the DSM-5 (SCID-5)^78^	Semi-structured interview	Semi-structured interview guide designed to be used by clinicians to assess for and diagnose mental disorders, including schizophrenia spectrum disorders, according to the DSM-5 diagnostic criteria.	Bourgou et al. (2016) Bartholomeusz et al. (2018) Vargas et al. (2019) Shi et al. (2020) Carey et al. (2021) El Ray et al. (2022) Shen et al. (2022)
Structured Interview for Prodromal Symptoms (SIPS)^79^	Semi-structured interview	Semi-structured interview guide designed to be used by clinicians to assess individuals in a pre-psychotic state. It includes ratings along four major symptom dimensions: positive, negative, disorganized, and general/affective symptoms.	Jalbrzikowski et al. (2012) Zhang et al. (2016) Vargas et al. (2019) Boldrini et al. (2020) Ilzarbe et al. (2021) Kong et al. (2021) Sardella et al. (2023)
Schizotypal Personality Questionnaire (SPQ)^80^	Self-report questionnaire	74-item self-report questionnaire designed to measure schizotypal traits. It has nine subscales, each related to one of three schizotypy dimensions: cognitive-perceptual, interpersonal, and disorganization. Items are rated on a Yes/No response format.	Salaminios et al. (2021) Salaminios et al. (2023)
Schizotypal Personality Questionnaire Brief (SPQ-B)^81^	Self-report questionnaire	Shortened 22-item version of the SPQ. It is comprised of three main subscales: cognitive-perceptual, interpersonal, and disorganization. Items are rated on a Yes/No response format.	Jahshan and Sergi (2007)
The Schizophrenia Proneness Instrument(SPI-A and SPI-CY) and Child^82^	Clinician-rated scale	Clinician-rated scale assessing the presence of 9 cognitive symptoms (COGDIS criterion) and 10 perceptive symptoms (COPER criterion) included in Basic Symptom criteria for CHR-P.	Salaminios et al. (2023)
Youth Self-Report (YSR)^83^	Self-report questionnaire	112-item self-report questionnaire designed to measure psychopathology in youth aged 12-17, including thought problems relevant for psychosis. Participants rate items on a three-point Likert scale indicating how true each statement is when applied to themselves.	Salaminios et al. (2021)

FEP: First Episode Psychosis, CHR-P: Clinical high-risk for psychosis; DSM-5 = Diagnostic and Statistical Manual of Mental Disorders, Fifth Edition.

Eighteen of the twenty-one measures used to assess mentalizing focused on the ability to infer other peoples’ mental states (ie, other-oriented mentalizing). Fifteen of these were task-based measures of ToM and three were empathy questionnaires. In addition, two self-report measures and one interviewer-rated scale of reflective functioning were used. Contrary to ToM tasks that only focus on inferences about others’ mental states, measures of reflective functioning are designed to capture both self- and other-oriented facets of mentalizing.^18^

Eight of the seventeen measures used to assess psychosis expression were self-report questionnaires, predominantly used across studies in community samples to measure premorbid signs of psychosis expression, including schizotypal personality traits, as well as persecutory and magical ideation. Five semi-structured diagnostic interview scales were used to clinically assess CHR-P criteria, or for a diagnosis of FEP and psychotic disorders. Four interviewer-rated scales were used to provide continuous measures of psychosis symptom severity.

### Quality Assessment

Table 4 displays the quality assessment of studies, scored according to the QualSyst.^20^ The mean total score across all studies was 0.84 (SD = 0.09), indicating that the overall study quality was good.

**Table 4.** Quality Assessment of Included Studies by Using QualSyst Rating Tool (Studies Listed According to Total Score).

Study	Item 1	Item 2	Item 3	Item 4	Item 8	Item 9	Item 10	Item 11	Item 12	Item 13	Item 14	Total Score
Jahshan and Sergi (2007)	2	1	1	2	1	2	1	0	1	2	2	0.68
Canli et al. (2015)	1	1	2	2	1	1	2	1	2	1	1	0.68
Ilzarbe et al. (2021)	2	1	2	2	1	1	1	1	1	2	1	0.68
Sardella et al (2023)	1	1	2	1	2	1	2	1	1	2	1	0.68
Bourgou et al. (2016)	2	1	2	2	2	1	1	2	1	2	1	0.77
Saglam et al. (2022)	2	1	2	2	1	1	2	1	2	2	1	0.77
Langdon et al. (2014)	2	1	2	2	2	1	2	0	1	2	2	0.77
Fernyhough et al. (2008)	2	1	1	2	2	2	2	0	2	2	2	0.82
Shi et al. (2020)	2	2	2	2	2	1	1	0	2	2	2	0.82
Zhang et al. (2016)	2	1	2	2	2	1	2	1	2	2	1	0.82
Thompson et al. (2012)	2	2	2	2	2	1	1	0	2	2	2	0.82
Carey et al. (2021)	2	2	2	2	2	2	1	0	2	2	1	0.82
Korver-Nieberg et al. (2013)	1	2	2	2	2	1	2	1	2	2	2	0.86
Barragan et al. (2011)	2	2	2	2	2	2	2	2	2	2	1	0.86
Jalbrzikowski et al. (2012)	2	2	1	2	2	1	2	1	2	2	2	0.86
El Ray et al. (2022)	2	2	2	2	2	1	2	1	1	2	2	0.86
Li et al. (2017)	2	1	2	2	2	1	1	2	2	2	2	0.86
Ilzarbe et al. (2019)	2	2	2	2	2	1	1	1	2	2	2	0.86
Vargas et al. (2019)	1	2	1	2	2	1	2	2	2	2	2	0.86
Kong et al (2021)	2	1	2	2	2	1	2	2	1	2	2	0.86
Bartholomeusz et al. (2018)	2	2	2	2	1	1	2	2	2	2	2	0.91
Salaminios et al. (2021)	2	2	2	1	1	2	2	2	2	2	2	0.91
Boldrini et al. (2020)	2	2	2	2	2	2	1	2	2	2	2	0.95
Shen et al. (2022)	2	2	2	2	2	1	2	2	2	2	2	0.95
Salaminios et al (2023)	2	2	2	2	2	2	2	2	1	2	2	0.95
Steenhuis et al. (2019)	2	2	2	2	2	2	2	2	1	2	2	0.95
Clemmensen et al. (2015)	2	2	2	2	2	2	2	2	2	2	2	1.0
**Item Mean**	**1.87**	**1.63**	**1.83**	**1.96**	**1.75**	**1.33**	**1.63**	**1.17**	**1.75**	**1.96**	**1.71**	**0.84**

Items 5-7 were excluded; 2 = Criteria met; 1 = Criteria partially met; 0 = Criteria not met; Maximum possible total score for each paper = 1.0.

The two highest rated items were items 4 (M = 1.96, SD = 0.20) and 13 (M = 1.96, SD = 0.20), demonstrating that nearly all studies reported sufficient demographic information and that study findings were reported in adequate detail.

The next highest rated items were items 1 (M = 1.87, SD = 0.34) and 3 (M = 1.83, SD = 0.38), indicating that most studies described the aims and hypotheses of their investigation sufficiently and that the sampling strategy and variables used were appropriate and well-described.

Item 11 was the lowest rated (M = 1.17, SD = 0.81) suggesting that most studies lacked sufficient information about the variance estimates of their outcomes. Item 9 was the next lowest rated item (M = 1.33, SD = 0.48), highlighting that small sample sizes were used, particularly in studies that assessed group differences in mentalizing. Overall, only nine of the twenty-six studies were rated as fully meeting QualSyst assessment criteria for an appropriate sample size for the study design used and outcomes tested.

### Narrative Synthesis

For the purposes of the narrative synthesis and to systematically examine the presence of mentalizing difficulties and any associations between mentalizing and psychosis expression at each stage of its developmental continuum during adolescence and young adulthood, the findings of the reviewed studies are grouped and presented according to study population (ie, community samples, CHR-P samples, FEP samples, and clinical psychosis samples).

### Studies in Community Samples

Seven cross-sectional studies included in this review assessed mentalizing and psychosis expression in community adolescent and young adult samples, yielding mixed evidence for their relationship. Four of these tested group-differences in ToM abilities between participants scoring high on measures of schizotypal traits or magical ideation to those with low scores on those measures, neither of which reported significant differences between the two. Jahshan and Sergi^21^ used a video-based ToM task to assess the capacity to infer emotions, sarcasm, and beliefs in others^61^ in an undergraduate student sample. No group-differences were identified across these mentalizing domains between those with high and those with low total scores in a measure of schizotypal personality traits.^81^ In line with these findings, Fernyhough et al.^22^ did not report group-differences in performance on picture-based ToM-task^47^ between undergraduate students scoring at the top and bottom 5% on a measure of schizotypy.^74^ In addition, no significant associations were found in the total sample between ToM and positive schizotypy, negative schizotypy, total schizotypy, or persecutory ideation.^76^ Canli et al.^27^ assessed mentalizing abilities in a sample of community adolescents through the use of two verbal ToM tasks^50,62^ and an empathy self-report questionnaire.^48^ Analyses did not show group-differences in ToM or self-reported empathy between young people who scored high and those with low scores in a measure of magical ideation.^72^ Similarly, Barragan et al.^23^ reported that community adolescents with low and those with high total scores on a measure of schizotypy^73^ showed comparable performance in a verbal ToM task.^60^

In contrast to studies examining group-differences in mentalizing performance, four cross-sectional studies that used correlation or regression analyses to test linear associations between mentalizing and psychosis expression reported significant associations between the two. More specifically, higher scores on measures of psychosis expression were associated with lower scores on task-based and self-report measures of mentalizing abilities, as well as higher scores on assessments of mentalizing difficulties. As reviewed above, Barragan et al.^23^ did not identify differences in verbal ToM between high and low scorers in a schizotypy measure. Importantly, however, in the total sample, lower ToM was significantly associated with higher scores on positive schizotypy. Clemmensen et al.^28^ examined mentalizing performance in a verbal- and picture-based ToM task^65^ and its association with nonclinical psychotic-like experiences^71^ in a large community cohort of young people aged 11-12 years. After statistically accounting for the effects of other psychosocial risk factors for psychosis, results showed that a “hypermentalizing” ToM pattern involving the overattribution of mental states to others was significantly associated with an increased likelihood to report psychotic experiences. In line with these findings, two studies that assessed self-reported mentalizing abilities in community samples using the Reflective Functioning Questionnaire (RFQ)^18^ identified significant associations with a range of psychotic features. Salaminios et al.^40^ reported significant associations between schizotypal trait features measured by the Schizotypal Personality Questionnaire (SPQ)^80^ and RFQ scores in a sample of community adolescents. Specifically, higher scores on the social anxiety and odd speech SPQ subscales were associated with increased uncertainty in utilizing mental states to understand ones’ own and others’ behaviors. Furthermore, higher scores on the odd speech SPQ subscale were associated with reduced certainty in understanding mental states. Finally, higher uncertainty in mental states was associated with self-reported psychosis-relevant thought problems.^83^ More recently, Salaminios et al.^45^ examined the associations between RFQ-measured mentalizing and the presence of early state signs of psychosis risk as captured by interviewer-assessed cognitive and perceptive symptoms^82^ in a community sample of adolescents and young adults. Data showed that increased uncertainty and reduced certainty in mental states were independently associated with the presence of cognitive symptoms included in CHR-P criteria.

Only two studies utilized longitudinal designs to assess the relationship between psychosis expression and mentalizing in community adolescent and young adult samples, yielding contrasting findings. Steenhuis et al.^34^ reported that mentalizing abilities assessed using a verbal- and picture-based ToM task^64^ at 12-13 years of age did not predict the frequency of self-reported psychotic experiences^69^ at 6-year follow-up. The study further compared a subgroup of participants with baseline ToM scores at the lowest 10% with the rest of the sample and found no significant differences in the frequency of self-reported psychotic experiences at follow-up. Contrary to these findings, Carey et al.^38^ found that participants who reported psychotic symptoms^71,78^ during adolescence or young adulthood exhibited significantly worse mentalizing performance in a picture-based ToM task^55^ at follow-up (ages 19-24) compared with participants who did not report any psychotic symptoms at baseline.

### Studies in CHR-P Samples

Four cross-sectional studies included in this review examined mentalizing abilities in CHR-P samples, the majority of which reported that adolescents and young adults identified as CHR-P exhibit worse mentalizing abilities compared with nonclinical controls. Vargas et al.^35^ compared a group of adolescents and young adults meeting CHR-P criteria^78,79^ with age-matched nonclinical controls on a verbal ToM task assessing explicit mental state reasoning and spontaneous/implicit mental state inferences.^58^ Study findings showed that the two groups did not differ in explicit ToM; however, the CHR-P group made significantly less spontaneous mental state inferences, suggesting impairments in implicit ToM abilities. Thompson et al.^24^ assessed differences in verbal and picture-based ToM task performance^47,62^ between adolescents meeting criteria for CHR-P,^68^ a group of FEP youth and aged-matched nonclinical controls. Results showed that the CHR-P group performed significantly worse in ToM compared nonclinical controls, while no group-differences were identified between the CHR-P and FEP groups. Kong et al.^41^ tested mentalizing abilities through a picture-based ToM task^63^ and a self-report empathy questionnaire^52^ in young adults meeting CHR-P criteria.^79^ In contrast to studies described above, no differences in ToM were observed between the CHR-P group and age-matched controls. Nonetheless, the two groups differed significantly in their self-reported empathic concern. Finally, Sardella et al.^46^ compared self-reported mentalizing abilities, assessed by the brief version of the RFQ,^56^ between a group of adolescents and young adults meeting CHR-P criteria^79^ and nonclinical controls. Results showed that the CHR-P group reported higher uncertainty in mental states and lower mentalizing certainty compared with the control group.

Three studies employed longitudinal designs to examine mentalizing in CHR-P adolescents and young adults, specifically as it pertains to the predictive value of baseline mentalizing abilities in distinguishing between CHR-P participants who transitioned to FEP from those that did not transition. Overall, their findings tentatively suggest that mentalizing dysfunction among CHR-P samples is prospectively associated with increased risk for transition to FEP. Zhang et al.^30^ compared facial emotion recognition^55^ between a CHR-P group^79^ and an age-, gender-, and education-matched nonclinical control group, showing that CHR-P adolescents and young adults exhibited significantly worse ToM performance. Within the CHR-P group, a trend-level difference in baseline ToM performance was found, with those who converted to FEP at 9-month follow-up exhibiting worse baseline ToM scores compared with those that did not convert. Similarly, Boldrini et al.^36^ showed that mentalizing difficulties, assessed using a narrative interviewer-rated measure of reflective functioning,^57^ significantly predicted conversion to FEP at 11-19 month follow-up in a sample of young adults identified as CHR-P.^79^ In addition, the CHR-P group showed significantly lower reflective functioning scores compared with a clinical control group. Finally, reflective functioning scores within the CHR-P group were negatively associated with attenuated psychotic symptoms including unusual thought content/delusional ideas, suspiciousness/persecutory ideas, and disorganized communication.

In contrast to the studies described above, Ilzarbe et al.^39^ did not identify baseline differences in mentalizing performance in a picture-based ToM task^55^ between adolescents meeting CHR-P criteria^79^ who transitioned to FEP at 6-18 month follow-up and those who did not. Furthermore, no baseline differences in ToM were identified between CHR-P adolescents and nonclinical controls. Importantly however, significant and trend-level increases in ToM performance with increasing age were observed during the study interval in nonclinical controls and CHR-P who did not transition to FEP, respectively, while this age-effect was not observed among CHR-P adolescents who transitioned to FEP.

### Studies in FEP Samples

Four cross-sectional studies examined mentalizing abilities in adolescent and young adult samples presenting with FEP. The majority of these found that FEP adolescents and young adults exhibit impaired mentalizing abilities. As reviewed above, Thompson et al.^24^ found that an adolescent FEP group performed significantly worse than age-matched nonclinical controls on verbal and a picture-based ToM tasks.^47,62^ Bartholomeusz et al.^32^ found that a group of young people diagnosed with FEP^67,68,77,78^ performed significantly worse than nonclinical youths on a verbal ToM task,^62^ while no significant group differences were observed in ToM performance on a picture-based task.^59^ Langdon et al.^26^ reported that youth presenting with FEP showed significantly worse performance compared with age-matched nonclinical controls in one verbal and two picture-based ToM tasks.^47,59,60^ The group-differences in ToM performance remained after statistically accounting for the effects of cognitive impairment. Contrary to these studies, Korver-Nieberg et al.^25^ did not report any significant differences in mentalizing between FEP adolescents and nonclinical control youths on a video-based ToM task.^54^ Additionally, in both clinical and control groups, no significant associations were found between ToM and measures of symptom severity.^69,70,75^

### Studies in Samples Diagnosed With Psychotic Disorders

Six studies assessed mentalizing abilities among adolescents and young adults diagnosed with psychotic disorders, all of which confirm the presence of mentalizing impairments among clinical samples. Shen et al.^44^ reported that young adults suffering with schizophrenia performed significantly worse on a picture-based emotion-recognition ToM task^55^ compared with age-matched nonclinical controls. In addition, the clinical group showed significantly lower emotion-recognition accuracy and significantly longer task response times compared with nonclinical controls. Shi et al.^37^ found that a group of adolescents diagnosed with schizophrenia showed significantly lower overall scores on a parent-reported empathy questionnaire^51^ compared with age-matched nonclinical youths. Post-hoc analyses showed that the two groups differed significantly in parental reports pertaining to their children’s cognitive empathy, while no significant differences were observed for affective empathy.

Bourgou et al.^29^ found that a group of adolescents suffering with schizophrenia performed significantly worse than nonclinical controls in a ToM task requiring them to accurately infer goal-directed movement patterns and mentalizing interactions between geometric shapes shown in a computer screen.^53^ Furthermore, compared with nonclinical controls, the clinical group exhibited a hypermentalizing ToM pattern by over-attributing mental states and goal-directed intentions to random movements.

Li et al.^31^ investigated mentalizing in adolescents diagnosed with schizophrenia with a verbal task^50^ testing participants’ ability to detect and understand social “faux pas” situations and a visual task^66^ assessing the ability to make first-order (eg, “she is thinking of…”) and second-order (eg, “she thinks that he thinks…”) inferences pertaining a character’s thoughts and feelings. Results showed that young people suffering with schizophrenia performed significantly worse across all the ToM domains tested compared with age-matched nonclinical controls. However, within the clinical group, no significant associations were found between ToM and the severity of positive or negative symptoms.^75^

El Ray et al.^42^ found that young people suffering with schizophrenia demonstrated significantly worse scores than nonclinical controls on two picture-based ToM tasks assessing emotion recognition from facial or eye expressions.^49,55^ Within the clinical group, worse performance on the facial emotion recognition task was significantly associated with greater severity of negative symptoms.^75,78^ Using the same eye expression task, Ilzarbe et al.^33^ found that young people diagnosed with psychotic disorders showed significantly worse emotion recognition performance than a group of nonclinical youths. Importantly, emotion recognition performance showed a positive significant association with age in the nonclinical group, but this age-effect was not evident in the clinical group.

Finally, Sağlam et al.^43^ compared mentalizing abilities between young people suffering with symptomatic schizophrenia, young people with remitted schizophrenia and nonclinical controls. Participants’ mentalizing performance was assessed through a verbal faux pas task^50^ and a picture-based emotion recognition task.^55^ In line with the studies reviewed above, data showed that the two clinical groups performed significantly worse than healthy controls on both tasks. Results further showed that when statistically accounting for the effects of age, education, and medication dosage, young people with symptomatic schizophrenia showed significantly lower emotion recognition scores compared with the remitted group.

## Discussion

To the best of our knowledge, this is the first systematic review and evaluation of existing research that explored mentalizing abilities across each stage of the psychosis continuum, specifically during the critical period spanning from adolescence to young adulthood. Overall, the review of findings suggests that mentalizing impairments are observable across the continuum of psychosis expression during adolescence and young adulthood, including prior to the onset of clinical illness.

First, within samples of community adolescents and young adults, the evidence-base linking mentalizing difficulties and premorbid psychotic manifestations (eg, schizotypal traits, psychotic experiences) appears mixed. Specifically, four cross-sectional studies reviewed did not report group-differences in ToM performance between participants with high scores on measures of schizotypal traits and magical ideation and those scoring low on these measures.^21–23,27^ In contrast, four cross-sectional studies testing linear associations between mentalizing and psychosis expression in adolescence did report significant associations for lower ToM with positive schizotypy,^23^ for hypermentalizing ToM with psychotic experiences,^28^ and for self-reported mentalizing difficulties with schizotypal trait features,^40^ psychosis-relevant thought problems,^40^ and cognitive symptoms included in CHR-P criteria.^45^ Thus, it is possible that among community adolescent samples, study designs involving extreme-group or median-split comparisons may not be sensitive enough to capture links between subtle mentalizing difficulties and nonclinical signs of psychosis expression.^84^ It must also be noted that most studies used task-based measures that test basic ToM abilities and are prone to ceiling effects amongst nonclinical participants,^84^ thus may not have accurately captured individual differences in mentalizing among community samples. In a similar vein, ToM tasks only capture the ability to understand others’ mental states and do not directly assess difficulties in reflecting on one’s own thoughts and feelings. It has previously been proposed that impairments in self-oriented mentalizing may play a role in the earliest premorbid stages of psychosis expression.^10,11^ Indeed, two studies that used a self-report measure of reflective functioning designed to also assess self-oriented aspects of mentalizing reported associations with a range of nonclinical psychotic features.^40,45^ Overall, methodological differences pertaining to study design and mentalizing assessment may account for the mixed cross-sectional findings in community samples.

Interestingly, findings from longitudinal research among community adolescents showed that although mentalizing was not prospectively associated to the development of psychotic symptoms,^34^ the expression of nonclinical psychotic symptoms in adolescence did prospectively account for mentalizing impairments in young adulthood.^38^ Previous research suggests that the developmental elaboration of mentalizing continues throughout adolescence and into adulthood^85,86^ and is facilitated by the widening of interpersonal relationships,^9^ along with structural and functional changes in key neural networks contributing to cerebral specialization of mentalizing processes.^87^ Thus, it remains possible that the pathophysiology of emerging psychosis during adolescent development may interfere with both the social learning opportunities and brain maturation processes linked with the normative elaboration of mentalizing. It is important for future studies to longitudinally assess the complex nature of associations linking early signs of emerging psychosis with the elaboration of mentalizing processes and how the two may interact to modulate risk for clinical psychosis in combination with other risk or protective factors.

Among adolescent and young adult CHR-P samples, the majority of cross-sectional studies using ToM tasks, as well as self-report and interviewer-rated measures of reflective functioning suggest the presence of generalized impairments in mentalizing. These findings lend empirical support to the notion that mentalizing difficulties do not represent epiphenomena of clinical illness severity and chronicity but are already present during the prodromal stage of psychosis and may contribute to its clinical progression.^15^ Indeed, a small amount of longitudinal research suggests that mentalizing impairments during adolescence and young adulthood may predict transition to FEP among CHR-P samples.^30,36^ While the specific causal mechanisms linking mentalizing difficulties to the development of clinical psychosis remain unclear, it has been suggested that the normative elaboration of mentalizing abilities during adolescence may exert a protective effect against transition to clinical illness among those who are at increased risk.^10^ Consistent with this hypothesis, one longitudinal study included in this review showed that although differences in ToM were not sufficient to predict conversion from CHR-P to FEP, age-related improvements in ToM were more pronounced among nonclinical controls and CHR-P individuals who did not transition to clinical illness compared with those who did.^61^

In line with findings from CHR-P samples, studies among adolescents and young adults presenting with FEP and with a previous diagnosis of psychotic disorders suggest that mentalizing impairments are present at the point of conversion and early course of the illness and appear to relate to symptomatic severity. It must be noted that findings from these studies were based on small sample sizes due to the low incidence rates of psychotic disorders in adolescence. Nonetheless, their findings are consistent with those in adult samples.^14,15^ Thus, this systematic review adds to the literature, suggesting that mentalizing impairments akin to those observed in adults suffering with psychotic disorders are also present among affected adolescents and may modulate early symptomatic and functional outcomes. Indeed, it has been shown that better mentalizing abilities among young people recovering from FEP are linked with an increased ability to adaptively cope with the illness and effectively navigate adolescent-specific developmental challenges pertaining to identity formation and the initiation of new peer relationships.^88^

While the review findings do not permit causal conclusions about the nature of associations linking mentalizing and psychosis expression, we have previously hypothesized that the two may dynamically impact upon each other across development to augment vulnerability for clinical illness.^89–91^ Specifically, among genetically predisposed individuals, the expression of premorbid and prodromal manifestations in adolescence (eg, social anxiety, suspiciousness/paranoid ideation) may inhibit the close interpersonal contact that normally sustains the capacity to monitor, understand, and reflect on one’s own and others’ mental states. Concurrently, failures to reflect on and regulate one’s own inner states along with difficulties in adaptively using mental states to understand others’ behaviors within interpersonal situations may exacerbate psychosis expression and increase social withdrawal, leading to clinically diagnosable presentations and undermining recovery processes in the early course of the illness. Beyond the use of longitudinal designs, future studies can test these assumptions by utilizing novel network approaches^92^ to examine dynamic and causal interrelationships between distinct symptomatic clusters, mentalizing domains, and other psychosocial risk factors for psychosis across each stage of its developmental continuum.

Overall, the findings of the systematic review suggest that mentalizing difficulties can be observed across the psychosis continuum in adolescence and young adulthood, particularly during the prodromal and clinical stages of expression, and may constitute empirically and clinically valid early intervention treatment targets. On this basis, the application of mentalization-based treatment (MBT)^11,93^ and other interventions with a mentalizing focus may be warranted to sustain resilience against transition to clinical illness in adolescents and young adults presenting with CHR-P and to support recovery among affected individuals.^89–91,94^ In line with this, recent outcome data from an MBT intervention with young people meeting CHR-P criteria or with a diagnosis of psychotic disorders showed clinically and statistically significant improvements across a range of symptomatic and functional domains.^95^ For young people presenting with trait-like psychotic manifestations, in accordance with clinical staging principles,^96^ mentalization-focused interventions, ranging from psychoeducation about mentalizing provided at the school and family levels to more intensive interventions within group or individual treatment settings, can be applied according to the level of need and impact of symptoms on the young person’s school functioning and peer-relationships.^5^

## Limitations and Conclusions

A number of methodological limitations associated with the reported studies need to be taken into account when considering the findings of the current review. First, significant variations in study design, including assessment and data analytic methods, precluded us from conducting a meta-analysis of the data. It must be noted that previous meta-analyses have reported significant differences in ToM between adult samples at different stages of the psychosis continuum, with CHR-P samples performing worse that community samples, but better than FEP and chronic psychosis samples.^15^ Second, the majority of reviewed studies used ToM tasks in which social situations were presented to participants either verbally (eg, listening to stories) or via pictures (eg, cartoon strips). Importantly however, ToM inferences made on the basis of pictorially or verbally presented material do not approximate the demands of real-life social situations, which also depend on the evaluation of contextual and physical cues as these naturalistically unfold during social interactions (eg, prosodic information, postural movements, eye contact). In a similar vein, the self-report measures used tend to assess mentalizing abilities in a decontextualized manner that may not take into account the inherently interactional context within which mentalizing unfolds.^97,98^ Third, studies among young people presenting with FEP and psychotic disorders were based on small sample sizes due to the low prevalence of clinical psychosis in adolescence, and thus, their findings need to be treated with caution. Finally, given that the majority of reviewed studies used cross-sectional designs, it remains difficult to establish causal inferences regarding the associations between mentalizing and psychosis expression.

Despite these limitations, this is the first systematic review to synthesize existing research on the links between mentalizing and psychosis expression during the critical developmental period spanning from adolescence to young adulthood. Overall, study findings highlight the relevance of mentalizing across the developmental continuum of psychosis. From a clinical standpoint, ineffective or dysfunctional mentalizing appears to be a valid clinical assessment and treatment target in the context of early intervention during adolescence and young adulthood.^5^

## Supplementary material

Supplementary material is available at https://academic.oup.com/schizophreniabulletin.

sbaf095_suppl_Supplementary_Material
