# Supplementary material for: Mentalizing Across the Psychosis Continuum in Adolescence and Young Adulthood: A Systematic Review and Narrative Synthesis
Source: Schizophr Bull. 2025 Jul 10;52(4):sbaf095. doi: 10.1093/schbul/sbaf095 (PMC13391650; doi:10.1093/schbul/sbaf095)
Supplement: sbaf095_suppl_Supplementary_Material [file sbaf095_suppl_supplementary_material.docx]

**Supplementary material**

**Search queries used for each database search**

| **PsycINFO** | ((mentaliz* or mentalis* or "theory of mind" or "reflective function*") and (psychosis or psychotic or schizo* or hallucin* or delusion* or "clinical high-risk" or "ultra-high risk" or "at-risk mental state") and (adolescen* or youth* or "young adult*" or "emerging adult*" or "young people" or student)).ab,ti,id. |
| --- | --- |
| **Embase** | ((mentaliz* or mentalis* or "theory of mind" or "reflective function*") and (psychosis or psychotic or schizo* or hallucin* or delusion* or "clinical high-risk" or "ultra-high risk" or "at-risk mental state") and (adolescen* or youth* or "young adult*" or "emerging adult*" or "young people" or student)).ab,ti,kf. |
| **MEDLINE** | ((mentaliz* or mentalis* or “theory of mind” or “reflective function*”) and (psychosis or psychotic or schizo* or hallucin* or delusion* or “clinical high-risk” or “ultra-high risk” or “at-risk mental state”) and (adolescen* or youth* or “young adult*” or “emerging adult*” or “young people” or student)).ab,ti,kf,kw. |
| **Web of science** | (mentaliz* OR mentalis* OR (“theory of mind”) OR (“reflective function*”)) (Topic) AND (psychosis OR psychotic OR schizo* OR hallucin* OR delusion* OR (“clinical high-risk”) OR (“ultra-high risk”) OR (“at-risk mental state”)) (Topic) AND (adolescen* OR youth* OR (“young adult*”) OR (“emerging adult*”) OR (“young people”) OR student) (Topic) |

**QualSyst items**

| **Criteria** | | **Yes (2)** | **Partial (1)** | **No (0)** | **N/A** |
| --- | --- | --- | --- | --- | --- |
| **1** | Question/objective sufficiently described? |  |  |  |  |
| **2** | Study design evident and appropriate? |  |  |  |  |
| **3** | Method of subject/comparison group selection or source of information/input variables described and appropriate? |  |  |  |  |
| **4** | Subject (and comparison group, if applicable) characteristics sufficiently described? |  |  |  |  |
| **5** | If interventional and random allocation was possible, was it described? |  |  |  |  |
| **6** | If interventional and blinding of investigators was possible, was it reported? |  |  |  |  |
| **7** | If interventional and blinding of subjects was possible, was it reported? |  |  |  |  |
| **8** | Outcome and (if applicable) exposure measure(s) well defined and robust to measurement misclassification bias? Means of assessment reported? |  |  |  |  |
| **9** | Sample size appropriate? |  |  |  |  |
| **10** | Analytic methods described/justified and appropriate? |  |  |  |  |
| **11** | Some estimate of variance is reported for the main results? |  |  |  |  |
| **12** | Controlled for confounding? |  |  |  |  |
| **13** | Results reported in sufficient detail? |  |  |  |  |
| **14** | Conclusions supported by the results? |  |  |  |  |
